# Diversity of Methicillin-Resistant Staphylococcus aureus Strains Isolated from Residents of 26 Nursing Homes in Orange County, California

**DOI:** 10.1128/JCM.01708-13

**Published:** 2013-11

**Authors:** Lyndsey O. Hudson, Courtney Reynolds, Brian G. Spratt, Mark C. Enright, Victor Quan, Diane Kim, Paul Hannah, Lydia Mikhail, Richard Alexander, Douglas F. Moore, Daniel Godoy, Cynthia J. Bishop, Susan S. Huang

**Affiliations:** Department of Infectious Disease Epidemiology, Imperial College London, London, United Kingdoma; School of Social Ecology and Division of Infectious Diseases, University of California Irvine School of Medicine, Irvine, California, USAb; Division of Infectious Diseases and Health Policy Research Institute, University of California Irvine School of Medicine, Irvine, California, USAc; Orange County Health Care Agency, Santa Ana, California, USAd

## Abstract

Nursing homes represent a unique and important methicillin-resistant Staphylococcus aureus (MRSA) reservoir. Not only are strains imported from hospitals and the community, strains can be transported back into these settings from nursing homes. Since MRSA bacteria are prevalent in nursing homes and yet relatively poorly studied in this setting, a multicenter, regional assessment of the frequency and diversity of MRSA in the nursing home reservoir was carried out and compared to that of the MRSA from hospitals in the same region. The prospective study collected MRSA from nasal swabbing of residents of 26 nursing homes in Orange County, California, and characterized each isolate by *spa* typing. A total of 837 MRSA isolates were collected from the nursing homes. Estimates of admission prevalence and point prevalence of MRSA were 16% and 26%, respectively. The *spa* type genetic diversity was heterogeneous between nursing homes and significantly higher overall (77%) than the diversity in Orange County hospitals (72%). MRSA burden in nursing homes appears largely due to importation from hospitals. As seen in Orange County hospitals, USA300 (sequence type 8 [ST8]/t008), USA100 (ST5/t002), and a USA100 variant (ST5/t242) were the dominant MRSA clones in Orange County nursing homes, representing 83% of all isolates, although the USA100 variant was predominant in nursing homes, whereas USA300 was predominant in hospitals. Control strategies tailored to the complex problem of MRSA transmission and infection in nursing homes are needed in order to minimize the impact of this unique reservoir on the overall regional MRSA burden.

## INTRODUCTION

Methicillin-resistant Staphylococcus aureus (MRSA) is a major cause of morbidity and mortality worldwide and has significant economic consequences (1–6). Residence in a nursing home, which typically provides long-term care for chronically ill and/or elderly people, is a well-established risk factor for MRSA carriage and infection (7–10), and MRSA carriage in nursing home residents is associated with increased mortality (11). Nursing homes represent a unique and important MRSA reservoir, as patients colonized with MRSA tend to introduce the organism into nursing homes from the hospital setting, and MRSA can also be transported back into hospitals and the community from the nursing home. A recent modeling study demonstrated that nursing homes play an important role in regional MRSA transmission dynamics (12). The reservoir represented by colonized patients is often large due to the high MRSA prevalence in nursing homes, sometimes higher than 30%, which increases the risk of MRSA transmission in these facilities (10, 13–15). Furthermore, once colonized, nursing home residents seem to carry the same MRSA strain for prolonged periods of time; asymptomatic colonization has been reported to last from 3 months to 3 years (16, 17). Studies suggest that multiple strains circulate within nursing homes (10, 16, 18).

Not only does the complex operational structure of nursing homes, which act as both a health care setting and a resident's home, make it difficult for standard MRSA control practices to be implemented, but a standardized MRSA control strategy for nursing homes is yet to be agreed on, largely due to the lack of studies aimed at identifying appropriate strategies (19). There is also a general dearth of studies, particularly regional ones, investigating the makeup of the nursing home MRSA reservoir. A study of 60 nursing homes in Belgium identified hospital care, comorbidities, and a lack of coordinated MRSA surveillance and control activities as risk factors for MRSA carriage in nursing home residents (20). A study of 32 nursing homes in northern Germany listed indwelling devices, wounds, preceding hospital admission, and high-grade resident care as risk factors for carriage (21). Both studies found that the predominant MRSA strains among nursing home residents were identical to those found in hospital inpatients, highlighting the need for synergistic infection control between nursing homes and hospitals (20, 21). A better understanding of the frequency and diversity of nursing home MRSA strains and predictors thereof will help to form strategies for minimizing MRSA transmission and infection in nursing homes, and thus reduce the impact of the nursing home MRSA reservoir on hospitals.

Assessing the extent to which traditionally community-associated MRSA (CA-MRSA) has penetrated the nursing home reservoir is also of interest. CA-MRSA has become increasingly dominant in recent years, and the MRSA clone USA300 in particular has several characteristics that may offer a selective advantage over the traditional health care-associated MRSA (HA-MRSA), including higher transmissibility and increased pathogenicity (22, 23). There is also growing evidence from several countries, particularly the United States, that community and health care reservoirs are mixing (24–30).

We conducted a prospective study of MRSA isolates in nursing home residents in a large U.S. metropolitan county to investigate the frequency and genetic diversity of MRSA in these facilities, and thus gain a better understanding of the nature of the nursing home MRSA reservoir.

## MATERIALS AND METHODS

### Study design.

We conducted a population-based prospective study of nares colonization isolates of MRSA from 26 nursing homes in Orange County, California. This study was approved by the Institutional Review Board of the University of California Regents, and a waiver of informed consent was granted.

### Isolate collection.

Carriage isolates of MRSA from unique residents were collected from participating nursing homes between January 2009 and April 2011. Each nursing home was instructed to swab the nares of 100 consecutive residents upon admission (within 3 days of arrival) and 100 residents on a single day (point prevalence screening) using bilateral nares swabs (BD Culture Swabs; Fisher Scientific). For nursing homes with a low bed turnover, fewer residents were screened (30–50). For nursing homes with an average length of stay in years, admission screening was not performed. Swabs were cultured for MRSA using selective medium (BD CHROMagar). MRSA strains were stored at −65°C in brucella broth containing 15% glycerol.

### Specimen data and nursing home characteristics.

Specimen data, including swab type (admission or point prevalence), swab day since admission, room type (shared or single resident room), and whether the swabbed resident had prior MRSA, were collected. Demographic, functional status, and comorbidity data for participating nursing homes were derived from the Centers for Medicare and Medicaid Services (CMS) Long Term Care Minimum Data Set for 2009 (http://www.resdac.org/MDS/data_available.asp; last accessed 10 April 2012) and included annual admissions and the percentage of residents with the following characteristics: <65 years of age; male; nonwhite; Hispanic; education less than high school level; admitted from a hospital; history of MRSA, diabetes, fecal incontinence, skin lesions, and medical devices (which included tracheostomy, ventilator, and dialysis devices).

### Laboratory methods and molecular typing.

All laboratory methods and molecular typing (*spa* typing, multilocus sequence typing [MLST], and SmaI pulsed-field gel electrophoresis [PFGE]) were performed as described previously (30). *spa* typing was performed on all collected isolates. MLST was performed on a subset of isolates (*n* = 138), and the combination of MLST and *spa* typing information was used to infer strain types and major U.S. MRSA clones (USA100, USA300, etc.). This subset included one isolate of each *spa* type so that all *spa* types were represented, and for the 10 most common *spa* types, one isolate from each of the hospitals in which these *spa* types were present. Isolates were selected by using a random number generator.

### Definitions.

Throughout this study the terms “traditionally CA-MRSA” and “traditionally HA-MRSA” were used to emphasize that, while certain clones were originally identified as community- or health care-associated clones, such clones may well be present and/or established in both settings today. We used this terminology in order to more clearly evaluate any mixing of MRSA reservoirs.

### Statistical analyses.

The median and interquartile range (IQR) were calculated for each variable. One- and two-sample z-tests for equality of proportions were conducted to compare the number of isolates belonging to *spa* types t008 and t002/t242 within each nursing home, the number of t002 and t242 isolates within each nursing home, and overall MRSA carriage at admission versus MRSA point prevalence. Simpson's index of diversity (1 − *D*) was used to estimate inter- and intra-nursing home genetic diversity of the MRSA strains collected, as well as the genetic diversity of the two major *spa* clonal complexes (*spa*-CCs) and genetic diversity among admission and point prevalence isolates. Simpson's index of diversity provides an unbiased measure of the probability of drawing two different *spa* types given the distribution of *spa* types in a sample (31). Confidence intervals (95% confidence intervals [95% CIs]) were calculated as described previously (32). For comparison of diversity indices, a significant difference (*P* < 0.05) was determined by nonoverlapping 95% CIs. χ^2^ tests compared the proportion of isolates belonging to *spa* types t008 and t002/t242 between nursing homes and between MRSA admission and point prevalence isolates. Pearson's correlation coefficients were computed to determine the relationships between nursing home variables and genetic diversity and between isolate variables and genetic diversity. Due to the small sample size of nursing homes (21 nursing homes included; of the 26 nursing homes, one did not isolate any MRSA, and four were excluded in this analysis, as they collected <10 MRSA isolates) and the number of potential predictor variables for genetic diversity, variables were considered for entry into a bootstrapped multiple linear regression model based on a combination of their correlation coefficient and current knowledge regarding their association with MRSA. Only variables with *P* < 0.1 in correlation tests were considered for the exploratory model. All statistical tests were performed using STATA (release 11, StataCorp 2009).

## RESULTS

Between January 2009 and April 2011, 3,806 nasal swabs were taken from residents of 26 nursing homes in Orange County, California, either on admission or for estimating MRSA point prevalence. Of these, 837 swabs (22%) isolated MRSA. The overall admission prevalence for the 26 nursing homes was 16%, and point prevalence was significantly higher at 26% (*P* < 0.001). One nursing home did not isolate any MRSA and was not considered further.

The majority of the 837 MRSA isolates were from point prevalence testing (68%), from residents with no history of MRSA (76%), and from residents sharing a room (95%). The median number of days from admission to swab collection was 53 (IQR, 4 to 265). A third of all admission swabs were collected at day 4 since some nursing homes could not swab earlier. Tables 1 and 2 give a summary overview of the 26 nursing homes and isolate characteristics.

**Table 1:** Summary of characteristics of the 26 participating nursing homes in Orange County, California

Characteristic	Median value (IQR value^*a*^)
Annual no. of admissions	263 (138–520)
% residents of <65 years of age	22.5 (4–40)
% male	42.2 (31.6–50.3)
% education less than high school level	23.9 (7.4–34.1)
% Hispanic residents	14.3 (3.7–23)
% nonwhite residents	15.7 (7.8–21.8)
% residents admitted from a hospital	81.9 (56.6–93.8)
% residents with diabetes	26.9 (22.7–42.1)
% residents with fecal incontinence	42.2 (26.4–54.8)
% residents with skin lesions	72.2 (50–86.5)
% residents with devices	2.2 (1.2–7.1)
% residents with MRSA history	11.5 (6–19)
MRSA admission prevalence	16 (11–22)
MRSA point prevalence	26.3 (16–34)
No. of *spa* types per nursing home	5 (4–8)

a IQR, interquartile range.

**Table 2:** Summary of characteristics of the 837 MRSA carriage isolates from nursing home residents in Orange County, California

Characteristic	No. of isolates (%)			
	Total MRSA	t008	t242	t002
Total	837	222 (26.5)	273 (32.6)	195 (23.3)
Admission	269 (32.1)	64 (23.8)	90 (33.5)	69 (25.7)
Point prevalence	568 (67.9)	158 (27.8)	183 (32.2)	126 (22.2)
Resident has history of MRSA	201 (24.0)	53 (26.4)	58 (28.9)	58 (28.9)
Resident living in shared room	795 (95.0^*a*^)	219 (27.5)	257 (32.3)	179 (22.5)

a 92.6% of all swabbed patients shared a room.

### *spa* typing and MLST.

Of the 837 MRSA isolates collected, 835 were *spa* typed. Two isolates could not be *spa* typed, as one did not grow upon culturing and a *spa* PCR product was not obtained from the other. Among the 835 MRSA isolates, 60 *spa* types were identified, including nine *spa* types (1.4% of all isolates) that did not match any known *spa* sequence. These novel *spa* sequences were automatically submitted to the Ridom SpaServer via the Ridom StaphType software and were assigned new *spa* types. One isolate that was nontypeable (NT) by *spa* typing was identical to *spa* type t002, except for two extra nucleotides in the third repeat, making the repeat 26 bp long and putting the *spa* coding region out of frame. This isolate was retested to confirm that the result was not due to a processing error, and the *spa* type sequence was submitted to Ridom for their records. A clinical MRSA isolate from a patient in a hospital in Orange County, California, was similarly NT, and others have reported *spa* repeats of unexpected length (J. Rothganger, Ridom GmbH, personal communication) (29). The three most common *spa* types were t242, t008, and t002, representing 83% of all isolates collected (Table 3). The remaining 57 *spa* types each represented 1.4% or less of all isolates (Table 3; see Table S1 in the supplemental material).

**Table 3:** Ten most frequently found *spa* types among MRSA isolates from residents of nursing homes in Orange County, California^*a*^

Rank	*spa* type	MLST^*b*^	Frequency	%	Cumulative %
1	t242	5	273	32.7	32.7
2	t008	8	222	26.6	59.3
3	t002	5	195	23.4	82.6
4	t127	474	12	1.4	84.1
5	t306	5	11	1.3	85.4
6	t088	105	10	1.2	86.6
7	t037	239	7	0.8	87.4
8	t024	8	6	0.7	88.1
9	t068	8	6	0.7	88.9
10	t548	5	6	0.7	89.6
	Other		87	10.4	100.0

a The total number of *spa* types was 60, including one nontypeable isolate. Simpson's index of diversity (1 − *D*) value was 77% (95% CI, 75% to 78%). This table is based upon the results of 835 MRSA isolates, since two isolates were nontypeable by *spa* typing.

b MLST, multilocus sequence type.

**Fig 1:**
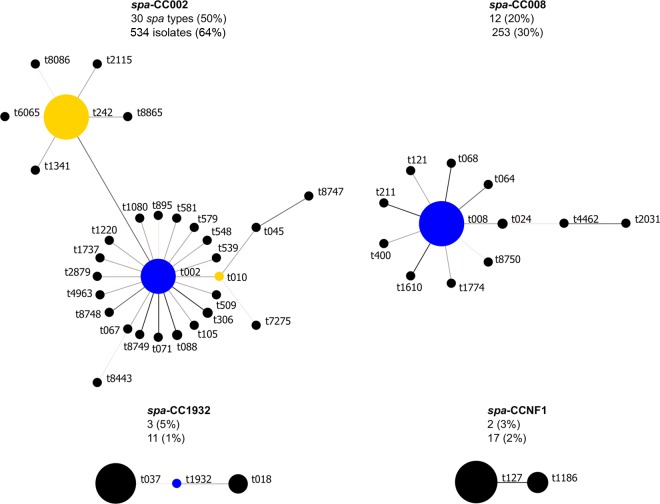
Relatedness of *spa* types among nursing home MRSA isolates. Relatedness was computed using the BURP (based upon repeat pattern) algorithm. Clusters of linked *spa* types correspond to *spa* clonal complexes (*spa*-CCs). *spa* types are clustered into a *spa*-CC when their repeat patterns differ by no more than 4 repeats. The BURP algorithm sums up “costs” (a measure of relatedness based on the repeat pattern) to define a founder score for each *spa* type in a *spa*-CC. The founder (blue node) is the *spa* type with the highest founder score in its *spa*-CC, and the subfounder (yellow node) is the *spa* type with the second highest founder score. *spa*-CC008 has founder t008, and *spa*-CCNF refers to a *spa*-CC with no founder. Each node represents a *spa* type. Node size represents the number of clustered strains that belong to that *spa* type. The shading of the branches represents the “costs” (similarities in repeat patterns) between two *spa* types; the darker the branch, the lower the cost (more similar repeat patterns).

BURP (based upon repeat pattern) analysis of the *spa* types clustered 94% of isolates into two large *spa* clonal complexes (*spa*-CCs) and 3% of isolates into two smaller *spa*-CCs (Fig. 1). Half of all *spa* types were clustered into *spa*-CC002 (predicted founder t002) and 20% into *spa*-CC008 (founder t008), including six novel *spa* types and one novel *spa* type, respectively. Using the BURP algorithm, *spa* types that differ from all other *spa* types in the sample by more than four repeats cannot reasonably be clustered into a *spa*-CC and are termed singletons. Ten *spa* types (17 isolates) were classed as singletons, including one novel *spa* type. Two isolates representing two *spa* types (t026 and t8606) were less than five repeats in length and were excluded from BURP analysis, because no reliable evolutionary history can be inferred from “short” *spa* types (33). The NT isolate could also not be included in the BURP algorithm. The estimated genetic diversity of MRSA in nursing homes in Orange County, California, using *spa* typing was high, at 77% (Table 3). The diversity of *spa* types among *spa*-CC008 isolates (1 − *D* = 23% [95% CI, 12 to 33%]) was significantly lower than the diversity among *spa*-CC002 isolates (1 − *D* = 60% [95% CI, 58 to 63%]).

To confirm strain types, 138 isolates were selected for MLST. Among the 15 unique sequence types (STs) identified, ST5 (54%) and ST8 (28%) were the most predominant, with the majority of isolates belonging to one of two major MLST CCs: CC5 (60%; five STs) and CC8/239 (29%; two STs) (Table 4). According to MLST, t008 isolates were ST8, and t002 isolates were ST5. t242 isolates were also identified as ST5 (Tables 3 and 4). While PFGE was not performed on the nursing home isolates, previous PFGE testing of Orange County MRSA isolates has shown that t008/ST8 isolates were the prototypic community clone USA300, and t002/ST5 and t242/ST5 isolates were predominantly the prototypic hospital clone USA100 (29, 30). *spa* type t242 differs from t002 by one *spa* repeat, as a result of a single-nucleotide difference. The NT *spa* isolate and one novel, singleton *spa* type were ST105, with seven of the other novel *spa* types being ST5 (78%) and one being ST8 (11%).

**Table 4:** Relatedness of STs of 138 nursing home MRSA isolates according to eBURST algorithm^*a*^

CC (no. of isolates)^*b*^	ST	Associated *spa* type(s)^*c*^
CC5 (83)	5	t002, t242, t306, 28 others
	105	t088, t002, t8444
	221	t002
	1011	t895
	1510	t242
CC8/239 (40)	8	t008, t024, 12 others
	239	t037
CC474/1900 (6)	474	t127, t1186
	1900	t127
Singletons (9)^*d*^	45	t026, t040, t736
	36	t018, t1932
	59	t437
	88	t5916
	188	t189
	217	t032

a eBURST (based upon related sequence types) algorithm.

b CC, clonal complex. All members of a CC share identical alleles at six of the seven loci with at least one other member of the CC.

c Only the three most common *spa* types are listed if more than three associated with that sequence type (ST).

d STs with allelic profiles that share less than six of their seven loci with all other STs in the data set.

### Nursing home differences.

**Fig 2:**
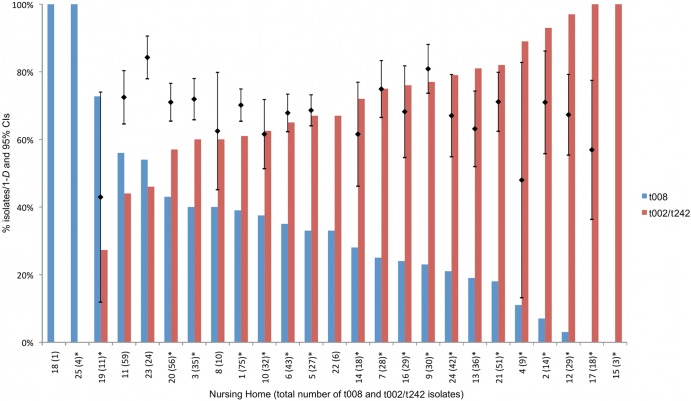
Relative proportion of isolates with *spa* type t008 versus *spa* types t002/t242, by nursing home. The presence of an asterisk after the total number of t008 and t002/t242 isolates indicates a significant difference at the 95% level in the relative proportion of isolates with *spa* type t008 and *spa* types t002/t242 at that nursing home. The black bars show the point estimates and 95% CIs of nursing home-specific genetic diversity expressed as Simpson's index of diversity (1 − *D*) of *spa* types (as a percentage). Diversity indices for nursing homes 15, 18, 22, and 25 were excluded from the figure, as these nursing homes had *spa* type data on less than 10 isolates. Diversity indices with nonoverlapping 95% CIs were considered significantly different (*P* < 0.05). The nursing home-specific proportions of t008 among all *spa* types have been previously reported (55).

Four nursing homes collected <10 MRSA isolates, and the MRSA genetic diversity could not be reliably estimated. For the 21 remaining nursing homes, the genetic diversity of MRSA ranged from 43% to 84% (Fig. 2). Genetic diversity was positively correlated with the percentage of residents admitted from a hospital (*r* = 0.52; *P* = 0.02), percentage of residents with diabetes (*r* = 0.57; *P* < 0.01), percentage of residents with skin lesions (*r* = 0.46; *P* = 0.03), MRSA admission prevalence (*r* = 0.50; *P* = 0.03), and MRSA point prevalence (*r* = 0.47; *P* = 0.03). Genetic diversity within nursing homes was negatively correlated with the percentage of residents under 65 years old (*r* = −0.57; *P* < 0.01) and the percentage of male residents (*r* = −0.43; *P* = 0.05).

The percentage of residents with skin lesions was positively correlated with the percentage of residents admitted from a hospital (*r* = 0.84; *P* < 0.001). The percentage of residents with skin lesions showed weaker correlation with genetic diversity and thus was not considered for entry into the bootstrapped linear regression model. Similarly, the percentage of male residents and the percentage of residents under 65 were highly correlated (*r* = 0.88; *P* < 0.001). Since age was more strongly correlated with genetic diversity than gender and differences in MRSA strain types have been observed between age groups in a previous study (30), the percentage of male residents was not considered for entry into the multiple regression model. Finally, point prevalence was highly correlated with admission prevalence (*r* = 0.74; *P* < 0.001); therefore, only admission prevalence was considered for regression model entry due to its slightly stronger correlation with genetic diversity. Only the percentage of residents aged 65 or over and the percentage of diabetic residents remained significant predictors of *spa* type genetic diversity in the exploratory regression model (coefficient = −0.22, bootstrap standard error = 0.06, normal-based 95% CI = −0.35 to −0.10, and *P* < 0.01 and coefficient = 0.41, bootstrap standard error = 0.10, normal-based 95% CI = 0.22 to 0.60, and *P* < 0.001, respectively).

Two nursing homes isolated t008 only, although the sample sizes were <10. For the other 23 nursing homes, the three most common *spa* types—t242, t008, and t002—accounted for 55 to 96% of isolates, showing that these *spa* types are consistently dominant across nursing homes in Orange County, California (see Table S1 in the supplemental material). *spa* type t242 was dominant in 12 nursing homes (36 to 63% of isolates).

The proportion of t008 (USA300) isolates compared to t002/t242 (USA100) isolates varied significantly between nursing homes (χ^2^ = 85.0; df = 24; *P* < 0.001) (Fig. 2). Two nursing homes (8%) had significantly more t008 (USA300) isolates, and 18 nursing homes (72%) had significantly more t002/t242 (USA100) isolates (*P* < 0.05). Of the t002/t242 isolates, 10 (40%) nursing homes collected significantly more t242 isolates, whereas 6 nursing homes (16.7%) collected significantly more t002 isolates (*P* < 0.05). Two nursing homes collected no t002/t242 isolates (each collected less than 10 MRSA isolates, and all were t008). The two nursing homes that isolated significantly more t008 (USA300) MRSA than t002/t242 (USA100) MRSA are therapeutic residential centers. Nursing homes with a higher percentage of residents admitted from a hospital had significantly lower percentages of t008 isolates (*r* = −0.61; *P* = 0.001).

### Admission versus point prevalence MRSA isolates.

The overall MRSA *spa* type genetic diversity was not significantly different among isolates collected at admission (1 − *D* = 76% [95% CI, 74 to 79%]) versus isolates collected during point prevalence testing (1 − *D* = 77% [95% CI, 75 to 79%]). Within individual nursing homes, no significant differences in MRSA *spa* type genetic diversity between admission and point prevalence testing were found (11 nursing homes were tested, and the 95% confidence intervals for admission and point prevalence diversity overlapped in all, indicating nonsignificance; the remaining 14 nursing homes did not isolate sufficient numbers, and thus, diversity indices could not be reliably estimated for these). No significant correlation was found between admission prevalence and the proportion of t008 isolates (*r* = 0.09; *P* = 0.73). The proportions of t008, t002, and t242 strain types were not significantly different between admission and point prevalence MRSA isolates (29%, 31%, and 40% among admission MRSA strains versus 34%, 27% and 39% among point prevalence MRSA strains, respectively; χ^2^ = 2.1; df = 2; *P* = 0.35).

## DISCUSSION

We conducted a prospective collection of carriage isolates of MRSA from 26 nursing homes in Orange County, California. The study investigated the frequency and genetic diversity of MRSA in these little-studied health care facilities to better inform nursing home-based infection control strategies. This is the first study to assess MRSA diversity in nursing homes at a population level and across a large region.

Countywide, nursing home carriage MRSA isolates were dominated in approximately equal proportions by three strains: MLST and previous PFGE testing of Orange County MRSA isolates identified these as the predominant, traditionally community-associated clone in the United States, USA300 (ST8/t008); the traditionally health care-associated clone USA100 (ST5/t002) and ST5/t242 isolates that likely represent a variant of USA100 that has become prevalent in health care facilities in Orange County, California (29, 30). ST5/t242 isolates were slightly more common than USA300 and USA100, representing a third of all Orange County nursing home carriage MRSA isolates. In Orange County hospitals, the same three strains dominated, but USA300 was the most common clone (29).

As in hospitals in Orange County, California, most *spa* types were closely related to either USA300 or USA100, creating two large, distinct *spa*-CCs (29). Of the two smaller *spa*-CCs, one represented strains of the traditionally CA-MRSA lineage MLST CC1, with most isolates typed as ST474/t127. ST474 is a single-locus variant (SLV) of ST1, and ST1/t127 is a common CA-MRSA strain in the United Kingdom (34). ST474/t127 isolates were also found among Orange County hospital inpatients, and t127 was also recently reported among U.S. isolates by Hudson et al. (29) and Tenover et al. (35). The other small *spa*-CC, *spa*-CC1932, represented traditionally HA-MRSA and included the epidemic clone USA200 (ST36/EMRSA-16) and the pandemic clone ST239.

The isolates that could not be assigned to a *spa*-CC included both traditionally health care- and community-associated MRSA strains. The traditionally health care-associated strains were USA600/Berlin clone (ST45) and ST217/t032, a SLV of ST22, the pandemic HA-MRSA clone EMRSA-15 that has also recently been reported in the community (36). The traditionally community-associated strains were USA1000 (ST59), ST188 (a double-locus variant of ST1 and ST474 reported sporadically in Australia and Asia [37–39]), and ST88, a clone closely related to CC1 that has been reported in several countries, particularly Nigeria, but has not been previously reported in the United States (40, 41).

It is clear that USA300 and USA100 dominate health care facilities in Orange County, California, in line with the MRSA picture seen nationwide. However, it would be interesting to investigate whether the USA100 t242 variant seen in this county is also more common than USA100 elsewhere in the United States. t242 has been reported sporadically and was found to be endemic in one hospital in Italy (42–45).

The overall genetic diversity of MRSA in nursing homes in Orange County, California, was significantly higher than that seen in hospitals in Orange County (29). The higher proportion of traditionally HA-MRSA strains present in nursing homes likely drives this, which could be a result of the high proportion of residents directly admitted to nursing homes from a number of different Orange County hospitals. Diversity was significantly lower among *spa*-CC008 isolates than *spa*-CC002 isolates. Since *spa*-CC002 is largely represented by USA100 and its close *spa* type relatives, it can be suggested that MRSA diversity in Orange County is driven by traditionally health care-associated strains.

In exploratory analyses, greater MRSA genetic diversity was significantly associated with older resident age and diabetic residents. Diabetic foot ulcers are a known risk factor for MRSA, and in particular traditionally HA-MRSA, with MRSA found to be present in 10 to 30% of diabetic wounds (46–49). Diabetic complications, such as neuropathy, osteomyelitis, and peripheral vascular disease, may result in a prolonged hospital stay, increasing the exposure of diabetic people to HA-MRSA (46). Older age is a well-established risk factor for HA-MRSA, as elderly patients tend to be sicker and require hospital treatment. Older age was a significant predictor of genetic diversity in hospitals in Orange County, California, and was associated with non-t008 strains in a study comparing adult and pediatric Orange County inpatients (29, 30).

In Orange County, California, traditionally community-associated strains are present in nursing homes but to a lesser extent than in hospitals (29), with the majority of nursing homes isolating significantly more MRSA traditionally associated with the health care setting. The vast majority of residents in this study were admitted to nursing homes directly from a hospital and thus were not recently exposed to the community MRSA reservoir, reducing the likelihood of isolating a traditionally community-associated strain at nursing home admission. While traditionally community-associated strains are increasing in hospital settings, it is likely that many hospitals have two incompletely mixed MRSA strain populations—a predominantly community-type MRSA reservoir among patients that came from and will return to the community, and a predominantly health care-type MRSA reservoir among patients that cycle into longer-term care settings. The nursing home resident demographic consists of older, sicker people who generally have a history of health care exposure and thus tend to have traditionally health care-associated strains, which are associated with more invasive infections and serious illness. In addition, the long-term care provided by nursing homes means that the resident turnover rate in these facilities is far lower than the patient turnover rate in hospitals. This results in a lower frequency of possible introductions of MRSA from outside the health care setting. Nonetheless, traditionally community-associated strains are penetrating the nursing home MRSA reservoir.

The significantly higher point prevalence of MRSA compared to admission prevalence may be due to the transmission within nursing homes of MRSA imported by residents upon admission. Previous studies of nursing homes in Orange County, California, found MRSA importation to be a strong predictor of MRSA prevalence, with burden and transmission driven by the number of residents with chronic illnesses or indwelling devices (15, 50).

A limitation of this study was that few individual-level characteristics were available, so facility-level data were primarily used. We also swabbed only a single site for MRSA. Although nares screening is thought to detect the majority of MRSA carriers (51, 52), anatomical sites such as the rectum and throat have been shown to be important in the detection of MRSA carriage (10, 53, 54). In using genetic diversity as the outcome, we reduced the sample size considerably, and thus multivariate analysis of predictor variables could only be exploratory. Also, the diversity indices estimated for *spa*-CC008 isolates versus *spa*-CC002 isolates may have been influenced by differing sample sizes (32).

This study was designed to assess the extent of MRSA reservoir mixing and to examine how this varies across nursing homes within a single, large U.S. county. The diversity of carriage MRSA isolates among nursing home residents, although heterogeneous between facilities, was significantly higher than the diversity among clinical MRSA isolates from hospital inpatients in the same county (77% versus 72%) (29). MRSA diversity in both hospitals and nursing homes appears to be driven by *spa* types closely related to those of USA100. The proportions of older, diabetic residents were significant predictors of nursing home MRSA diversity. When combining data from all nursing homes, we found that t002/t242 (USA100) and t008 (USA300) isolates dominated this setting, with ST5/t242 the most prevalent clone. USA300 was the second most common clone isolated from the nares of nursing home residents, suggesting substantial penetration of traditionally community-associated strains into the nursing home reservoir, but to a lesser extent than seen in hospitals, where USA300 was predominant. Nursing home MRSA burden appeared to be largely due to importation of diverse strains from hospitals and subsequent transmission of these imported strains, leading to high MRSA point prevalence. Nursing homes therefore represent a significant reservoir for MRSA, and as such, a consensual, regionally implemented control strategy tailored to this unique setting is required. However, due to the complexity of MRSA control in these facilities, further studies evaluating the contribution of nursing homes to regional MRSA transmission are needed before developing such a strategy.

## Supplementary Material

Supplemental material
