# Supplementary material for: Diversity of Methicillin-Resistant Staphylococcus aureus Strains Isolated from Residents of 26 Nursing Homes in Orange County, California
Source: J Clin Microbiol. 2013 Nov;51(11):3788–95. doi: 10.1128/JCM.01708-13 (PMC3889768; doi:10.1128/JCM.01708-13)
Supplement: Supplemental material [file supp_51_11_3788__index.html]

Supplemental material 

# Diversity of Methicillin-Resistant Staphylococcus aureus Strains Isolated from Residents of 26 Nursing Homes in Orange County, California

## Supplemental material

**Files in this Data Supplement:**

- Supplemental file 1 -

  Table S1 (*spa* type frequencies by nursing home for the 835 carriage MRSA isolates collected from 25 nursing homes in Orange County, CA)

  PDF, 62K
