# Supplementary material for: Diversity of Methicillin-Resistant Staphylococcus aureus Strains Isolated from Residents of 26 Nursing Homes in Orange County, California
Source: J Clin Microbiol. 2013 Nov;51(11):3788–95. doi: 10.1128/JCM.01708-13 (PMC3889768; doi:10.1128/JCM.01708-13)
Supplement: Supplemental material [file JCM.01708-13_zjm999092928so1.pdf]

Table S1. *spa* type frequencies by nursing home for the 835 carriage MRSA isolates collected from 25 nursing homes in Orange County, California.

Nursing Home	*spa* type	Frequency	%
1	t002	30	36.6
	t008	29	35.4
	t242	16	19.5
	t018	2	2.4
	t037	1	1.2
	t2031	1	1.2
	t437	1	1.2
	t4963	1	1.2
	t895	1	1.2
	Total	82	100.0
2	t242	8	44.4
	t002	5	27.8
	t008	1	5.6
	t189	1	5.6
	t2049	1	5.6
	t548	1	5.6
	NT	1	5.6
	Total	18	100.0
3	t008	14	35.9
	t002	12	30.8
	t242	9	23.1
	t189	2	5.1
	t067	1	2.6
	t400	1	2.6
	Total	39	100.0

4	t002	7	70.0
	t008	1	10.0
	t1610	1	10.0
	t242	1	10.0
	Total	10	100.0
5	t242	10	35.7
	t008	9	32.1
	t002	8	28.6
	t1341	1	3.6
	Total	28	100.0
6	t002	18	40.0
	t008	15	33.3
	t242	10	22.2
	t1610	1	2.2
	t8606	1	2.2
	Total	45	100.0
7	t242	13	38.2
	t002	8	23.5
	t008	7	20.6
	t8750	2	5.9
	t037	1	2.9
	t088	1	2.9
	t1341	1	2.9
	t211	1	2.9
	Total	34	100.0
8	t242	6	50.0
	t008	4	33.3
	t1341	1	8.3
	t736	1	8.3

	Total	12	100.0
9	t242	19	35.9
	t306	8	15.1
	t008	7	13.2
	t068	6	11.3
	t002	4	7.6
	t8748	2	3.8
	t024	1	1.9
	t026	1	1.9
	t127	1	1.9
	t189	1	1.9
	t548	1	1.9
	t6065	1	1.9
	t723	1	1.9
	Total	53	100.0
10	t242	17	50.0
	t008	12	35.3
	t002	3	8.8
	t045	1	2.9
	t2115	1	2.9
	Total	34	100.0
11	t008	33	45.2
	t002	13	17.8
	t242	13	17.8
	t088	5	6.9
	t037	2	2.7
	t306	2	2.7
	t539	2	2.7
	t010	1	1.4
	t024	1	1.4

	t105	1	1.4
	Total	73	100.0
12	t242	19	50.0
	t002	9	23.7
	t1774	5	13.2
	t008	1	2.6
	t121	1	2.6
	t306	1	2.6
	t8747	1	2.6
	t8865	1	2.6
	Total	38	100.0
13	t242	20	52.6
	t002	9	23.7
	t008	7	18.4
	t548	1	2.6
	t8749	1	2.6
	Total	38	100.0
14	t002	12	54.6
	t008	5	22.7
	t010	4	18.2
	t242	1	4.6
	Total	22	100.0
15	t002	3	75.0
	t1932	1	25.0
	Total	4	100.0
16	t242	19	51.4
	t008	7	18.9
	t002	3	8.1

	t5916	3	8.1
	t071	2	5.4
	t032	1	2.7
	t509	1	2.7
	t579	1	2.7
	Total	37	100.0
17	t242	15	62.5
	t002	3	12.5
	t024	3	12.5
	t8086	2	8.3
	t127	1	4.2
	Total	24	100.0
18	t008	1	100.0
	Total	1	100.0
19	t008	8	72.7
	t002	2	18.2
	t242	1	9.1
	Total	11	100.0
20	t008	24	38.7
	t002	19	30.7
	t242	13	21.0
	t127	2	3.2
	t037	1	1.6
	t040	1	1.6
	t1080	1	1.6
	t1220	1	1.6
	Total	62	100.0
21	t242	30	46.9

	t002	12	18.8
	t008	9	14.1
	t127	7	10.9
	t088	2	3.1
	t045	1	1.6
	t2879	1	1.6
	t509	1	1.6
	t581	1	1.6
	Total	64	100.0
22	t002	2	25.0
	t008	2	25.0
	t242	2	25.0
	t548	1	12.5
	t8444	1	12.5
	Total	8	100.0
23	t008	13	29.6
	t002	7	15.9
	t1186	5	11.4
	t242	4	9.1
	t4462	4	9.1
	t037	2	4.6
	t548	2	4.6
	t5916	2	4.6
	t064	1	2.3
	t088	1	2.3
	t2229	1	2.3
	t8443	1	2.3
	Total	43	100.0
24	t242	27	51.9
	t008	9	17.3

	t002	6	11.5
	t045	2	3.9
	t018	1	1.9
	t024	1	1.9
	t088	1	1.9
	t127	1	1.9
	t1737	1	1.9
	t400	1	1.9
	t7275	1	1.9
	Total	51	100.0
25	t008	4	100.0
	Total	4	100.0
